# Specific microRNA signatures responsible for immune disturbance related to hip fracture in aged rats

**DOI:** 10.1186/s13018-018-0721-5

**Published:** 2018-01-22

**Authors:** Xiaobin Chen, Jianzheng Zhang, Zhi Liu, Simeng Zhang, Tiansheng Sun

**Affiliations:** Institute of Orthopaedics, Chinese PLA Army General Hospital, Beijing, 100700 People’s Republic of China

**Keywords:** Hip fracture, Elderly, Immune disturbance, MiR-130a-3p, Interferon regulatory factor-1, Sphingosine-1-phosphate receptor 1

## Abstract

**Background:**

Hip fracture is commonly associated with an overwhelming inflammatory response, which may lead to high rates of morbidity and mortality in the elderly. MicroRNAs (miRNAs) play important roles in the functions of immune system. However, the association between miRNA dysregulation and immune disturbance (IMD) related to elderly hip fracture is largely unknown.

**Methods:**

In this study, microarray profiling was carried out to evaluate the differential expression patterns of miRNAs in plasma of the aged hip fracture rats with IMD, those without IMD, and normal aged rats, followed by validation using quantitative real-time reverse transcription polymerase chain reaction (qRT-PCR). Genes and signaling pathways of the dysregulated miRNAs related to elderly hip fracture-induced IMD were investigated in silico using Gene Ontology and analysis of Kyoto Encyclopedia of Genes or Genomes.

**Results:**

Dead or moribund rats with hip fracture exhibited significantly reduced TNF-α/IL-10 ratio compared with healthy controls and other hip fracture rats, which were therefore named as hip fracture rats with IMD. Seven serum miRNAs in hip fracture rats with IMD were significantly downregulated. qRT-PCR and in silico analysis revealed that miR-130a-3p likely participated in regulating the hip fracture-induced IMD. Furthermore, Western blot experiment demonstrated that in lung tissue, the reduction of miR-130a-3p was accompanied with the increase of the protein expression of interferon regulatory factor-1 (IRF1) and sphingosine-1-phosphate receptor 1 (SIPR1).

**Conclusions:**

miR-130a-3p desregulation may be associated with elderly hip fracture-induced IMD, which might act as a new potential biomarker for the diagnosis and prognosis of elderly hip fracture-induced IMD and a potential therapeutic target as well.

## Background

Hip fracture remains a leading cause of excessive morbidity and mortality among old people [1, 2]. Particularly, mortality associated with hip fracture is over 20% within 6 months [2]. Hip fracture becomes one of the most serious health care burdens affecting old people [3].

Trauma triggers an inflammatory response that can result in additional organ damage and even multi-organ failure [4]. The tumor necrosis factor-α (TNF-α) and interleukin-10 (IL-10) play an important regulatory role in the course of inflammatory responses [5–7]. The balance between TNF-α and IL-10 is important for the immune homeostasis maintenance, and the dysregulation of the TNF-α/IL-10 ratio might be predictive of complications in patients with inflammatory diseases [8]. Hip fracture is a common trauma in the elderly, which induces a state of inflammation. Hip fracture is reportedly associated with elevated systemic pro-inflammatory function [2, 6, 7, 9–11] and is thus accompanied with postoperative liver and lung dysfunction [2, 6, 7, 10, 12].

MiRNAs, small non-coding 22-nucleotide RNA molecules, involve in many biological and pathological processes such as tissue formation, cancer development, diabetes, neurodegenerative diseases, and cardiovascular diseases [13]. Particularly, it has been shown that some microRNAs (miRNAs) (e.g., miR-155, miR-146, miR-150) control the development and responses of the immune system [14]. However, there is little understanding on which miRNAs participate in the immune disturbance (IMD) related to hip fracture. In this study, we characterized and validated the dysregulated expression patterns of miRNAs related to hip fracture-induced IMD in rats by using microarray profiling, as well as analyzed genes and signaling pathways related to these dysregulated miRNAs using bioinformatics tools. This study might provide a new potential biomarker for the diagnosis and prognosis of IMD related to hip fracture in aged patients and a potential therapeutic target as well.

## Methods

### Experimental animals

Male Sprague Dawley (SD) rats were purchased from Beijing Vital River Laboratory Animal Technology Co., Ltd. (Beijing, China) and maintained on a 12:12 h light/dark cycle with free access to food and water. Animals were monitored every 3 days to check the status of movement and feeding. A total of 60 male SD rats, aged 22–23 months, defined as aged [15], and weighted 460–570 g, were used in this study. Rats were randomly divided into normal group (subject to anesthesia only, *n* = 15) and hip fracture group (subject to operation, *n* = 45), respectively. The experiment was designed to last for 72 h. The plasma of rats was collected at 24, 48, or 72 h after the hip injury. At 72 h, rats were sacrificed by intraperitoneal injection of phenobarbital, and the lung tissues were collected and stored at − 80 °C until use. This study was approved by the Institutional Committee of Animal Care and Usage of the Chinese PLA Army General Hospital (Beijing, China).

### Hip fracture model

The method was described previously by Zhang et al. [6]. In brief, rats were anesthetized by an intraperitoneal injection of xylazine (25 mg/kg) and ketamine (75 mg/kg) and were then placed on the base of a blunt guillotine ramming apparatus in a prone position with one rear leg immobilized by a rubber band to a screw. The proximal femur was identified and marked under the guidance of a C arm fluoroscopy (PLX112D, Siemens, Germany). A blunt guillotine with a weight of 500 g was dropped on it, with an average drop height of 14 cm. The force of the descending weight resulted in a unilateral closed proximal femoral fracture. After the modeling procedure, rats were put back in the feeding room to bind the injury site to relieve the pain and freely get access to food and water.

Rats in the normal group were anesthetized but were not subject to the following treatment.

### Analysis of serum TNF-α and IL-10

Blood samples were harvested from rats at 24, 48, and 72 h after the hip fracture injury. Serum concentrations of TNF-α and IL-10 were determined using an enzyme-linked immunosorbent assay kit (R&D, Minneapolis, MN, USA) according to the manufacturer’s instruction.

### MiRNA microarray

Three rats were randomly selected from each group, and the total RNA was isolated using an SLNco Total RNA Isolation kit (SLNco, Shanghai, China) in accordance with the manufacturer’s instructions. The quantity of RNA was determined using a Nanodrop 2000 spectrophotometer (Thermo Scientific, Wilmington, NC, USA). The concentration of RNA was assessed with an Agilent 2100 Bioanalyzer (Agilent, Santa Clara, CA, USA). Triplicate samples were for miRNA microarray using Affymetrix GeneChip MiRNA 4.0 Array (Affymetrix, Santa Clara, CA, USA). Microarray data were analyzed by Origene software (Origene, Beijing, China). Differentially expressed miRNAs were identified according to a fold-change of > 2 or < 0.5.

### Algorithm analyses

To identify possible mRNA targets and functions of the differentially expressed miRNAs, three different in silico analyses were performed, including Gene Ontology (GO), pathway analysis with Kyoto Encyclopedia of Genes or Genomes (KEGG), and the interaction analysis of miRNAs with genes using the Sanger miRNA database.

### RNA isolation and quantitative real-time reverse transcription polymerase chain reaction analysis

The differentially expressed miRNAs identified by microarray were validated through quantitative real-time reverse transcription polymerase chain reaction (qRT-PCR) analysis using an iQ5 real-time PCR detection system (Bio-Rad, Hercules, CA, USA) and the SYBR Premix Ex Taq™ kit (TaKaRa, Otsu, Shiga, Japan). Related primers were purchased from Dingguochangsheng Biotech. (Beijing, China), which were shown in Table 1.

**Table 1:** The primer sequences for miRNAs

miRNA	Primer sequence 5′—3′
miR-150-5p	F: CGCCAGGGTTTTCCCAGTCACGACTCTCCCAACCCTTGTACCAGT
	R: CGCGAGGAGAGAATTAATACGACTCAGTATACGCGCACTGGT
miR-130a-3p	F: CGCCAGGGTTTTCCCAGTCACGACCAGTGCAATGTTAAAAGGGCAT
	R: CGCGAGGAGAGAATTAATACGACTCAGTATACGCGATGCCCT
miR-143-3p	F:CGCCAGGGTTTTCCCAGTCACGACTGAGATGAAGCACTGTAGCTCA
	R:CGCGAGGAGAGAATTAATACGACTCAGTATACGCGTGAGCTA
miR-223-3p	F:CGCCAGGGTTTTCCCAGTCACGACTGTCAGTTTGTCAAATACCCC
	R:CGCGAGGAGAGAATTAATACGACTCAGTATACGCGGGGGTAT
miR-125b-1-3p	F:CGCCAGGGTTTTCCCAGTCACGACACGGGTTAGGCTCTTGGGAGCT
	R:CGCGAGGAGAGAATTAATACGACTCAGTATACGCGAGCTCCC
miR-6324	F:CGCCAGGGTTTTCCCAGTCACGACTCAGTAGGCCAGACAGCAAGCAC
	R:CGCGAGGAGAGAATTAATACGACTCAGTATACGCGGTGTTGC
U6	F:CTCGCTTCGGCAGCACA
	R:ACGCTTCACGAATTTGCGT

### Prediction of the potential target genes of miRNA

The potential target genes were predicted using Target Scan software (http://www.targetscan.org/vert_70/).

### Western blotting

Lung tissues were collected from the sacrificed rats, grinded and sonicated at 4 °C in lysis buffer containing 50 mM Tris (pH 7.5), 1 mM EDTA, 1% NP-40, 150 mM KCl, 2 mM NaF, 4 mM sodium orthovanadate, 0.2 mM Na_4_P_2_O_7_, 0.2 mM β-glycerol phosphate, and EDTA-free protease inhibitor mix (Roche, Basel, Switzerland). Protein concentration of the lysate was measured, and proteins were separated by standard SDS-PAGE using a 10% separating gel. A mouse anti-interferon regulatory factor-1 (IRF1) monoclonal antibody (1: 1000, Abcam, Cambridge, MA, USA) and rabbit anti-sphingosine-1-phosphate receptor 1 (SIPR1) monoclonal antibody (1:1000, Abcam) were used as primary antibodies. Goat or rabbit anti-mouse monoclonal antibody conjugated with horseradish peroxidase (Beijing Dingguochangsheng Biotech.) was used as the secondary antibody. The membrane was developed using the enhanced chemiluminescence method. Protein of each blot on the membrane was quantified based on the analysis of grayscale intensity using Quantity one software (Bio-Rad), which was normalized to β-tubulin.

### Statistical analysis

Data were analyzed using SPSS software Version 18.0 (SPSS, Chicago, IL, USA). Data were presented as mean ± standard error of the mean, and data at each time point were compared between all the groups using one-way analysis of variance with the following post hoc Tukey test. *P* < 0.05 was considered statistically significant.

## Results

### Analysis of serum cytokines after hip fracture

**Fig. 1:**
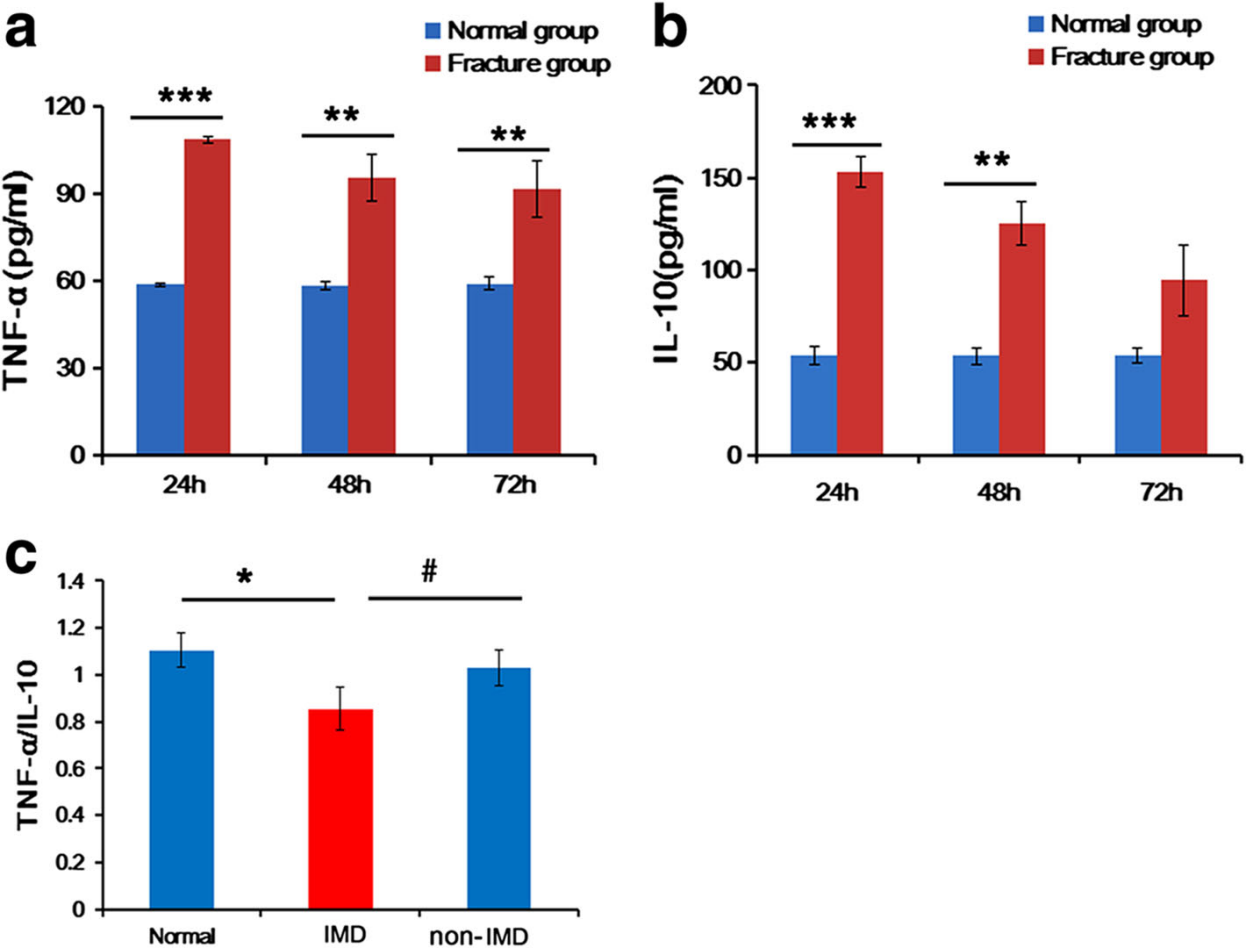
Serum levels of TNF-α, IL-10, and TNF-α/IL-10 ratio in hip fracture versus normal rats. **a**, **b** Serum level of TNF-α (**a**) and IL-10 (**b**) in hip fracture versus normal rats. **P < 0.01, ***P < 0.001 compared with normal rats. **c** Serum TNF-α/IL-10 ratio in normal, immune disturbance (IMD), and non-IMD rats. *P < 0.05 for comparison between IMD and normal rats, and ^#^*P* < 0.05 for comparison between IMD and non-IMD rats. *N* = 15 at each time point

To check the status of immune response associated with hip fractures, we determined the serum TNF-α and IL-10 levels as well as the serum TNF-α/IL-10 ratio in rats at 24, 48, or 72 h after the hip fracture. The serum levels of both TNF-α and IL-10 in hip fracture rats were gradually decreased after the operation, and the levels at 24, 48, or 72 h except that of serum IL-10 at 72 h were significantly higher than in normal rats (*P* < 0.01, Fig. 1a, b). 13.3% (6 of 45) of hip fracture rats were dead or became moribund at 72 h after hip fracture, and simultaneously, the serum TNF-α/IL-10 ratio in these rats was significantly downregulated compared with that in normal rats and other hip fracture rats (Fig. 1c). The reduction of TNF-α/IL-10 ratio indicates a status of IMD. Therefore, these dead and moribund rats with hip fracture were named as IMD rats and other hip fracture rats as non-IMD rats.

### MiRNA dysregulation signature in hip fracture-induced IMD

**Fig. 2:**
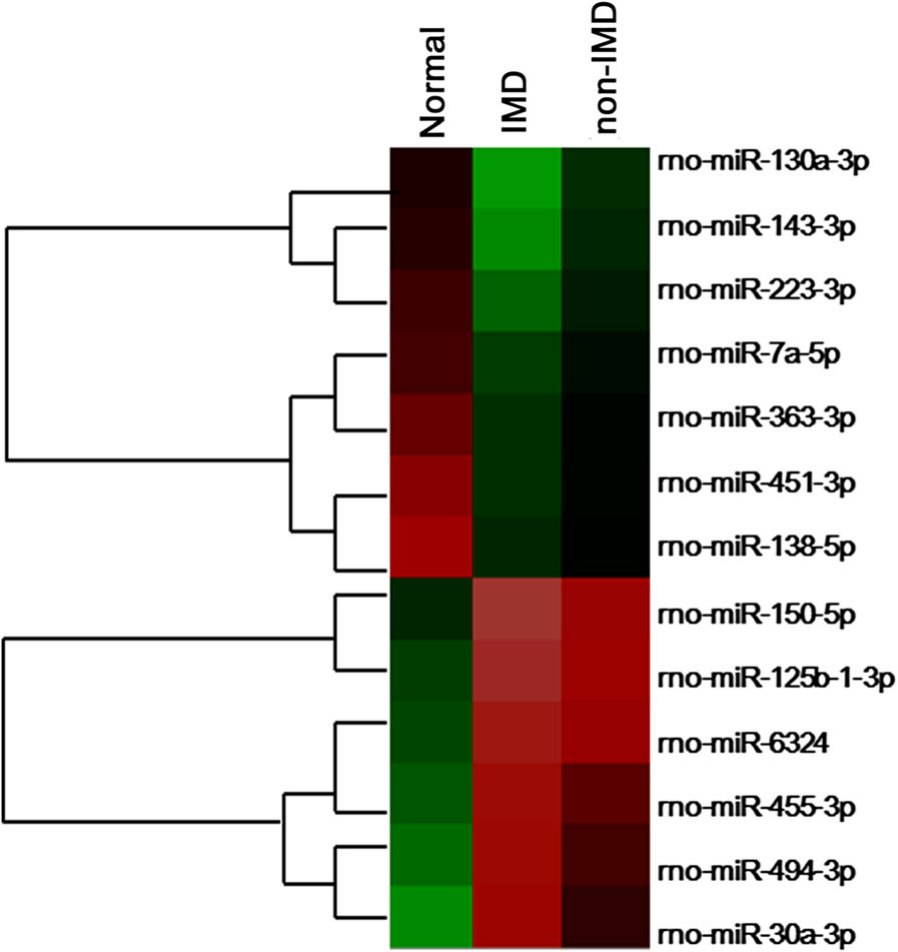
Hierarchical clustering analysis of the 13 differentially expressed miRNAs

To investigate the roles of miRNAs in the regulation of the hip fracture-induced IMD, miRNA microarray analysis was performed in serum samples of normal, IMD, and non-IMD rat group rats, three rats per each group. Thirteen differentially expressed miRNAs were identified in serum of IMD rats, including 6 with upregulated expression and 7 with down-regulated expression (Table 2). Through hierarchical cluster analysis, the 13 differentially expressed miRNAs among the normal, IMD, and non-IMD rats were normalized and generated a heat map (Fig. 2).

**Table 2:** Significantly differentially expressed miRNAs in serum of hip fracture rats with immune disturbance

Types of miRNAs	Function	Fold-change	*P* value
rno-miR-130a-3p	Inhibited cell proliferation, invasion, and migration	− 57.43	0.0226
rno-miR-143-3p	Inhibited cell proliferation and migration/invasion	− 56.63	0.0149
rno-miR-223-3p	An oncogene or tumor suppressor gene	− 49.46	0.0249
rno-miR-7a-5p	Inhibited cell proliferation	− 37.7	0.203
rno-miR-363-3p	Inhibited cell proliferation	− 28.08	0.0828
rno-miR-451-3p	Regulated tumor cell migration and cancer progression	− 25.15	0.0723
rno-miR-138-5p	Regulated cell radiation sensitivity	− 19.85	0.0157
rno-miR-150-5p	EV release from cardiac cells	3.92	0.0184
rno-miR-125b-1-3p	Respiratory electron transport, ATP synthesis by chemiosmotic coupling, and heat production by uncoupling proteins	2.87	0.062
rno-miR-6324	Inhibited sphingomyelin metabolism	2.83	0.718
rno-miR-455-3p	Inhibited cell proliferation	2.71	0.0448
rno-miR-494-3p	Inhibited cell proliferation	2.69	0.0871
rno-miR-30a-3p	Inhibited cell autophagy, migration, and invasion	2.50	0.09

### Validation of the differentially expressed miRNAs by qRT-PCR

**Fig. 3:**
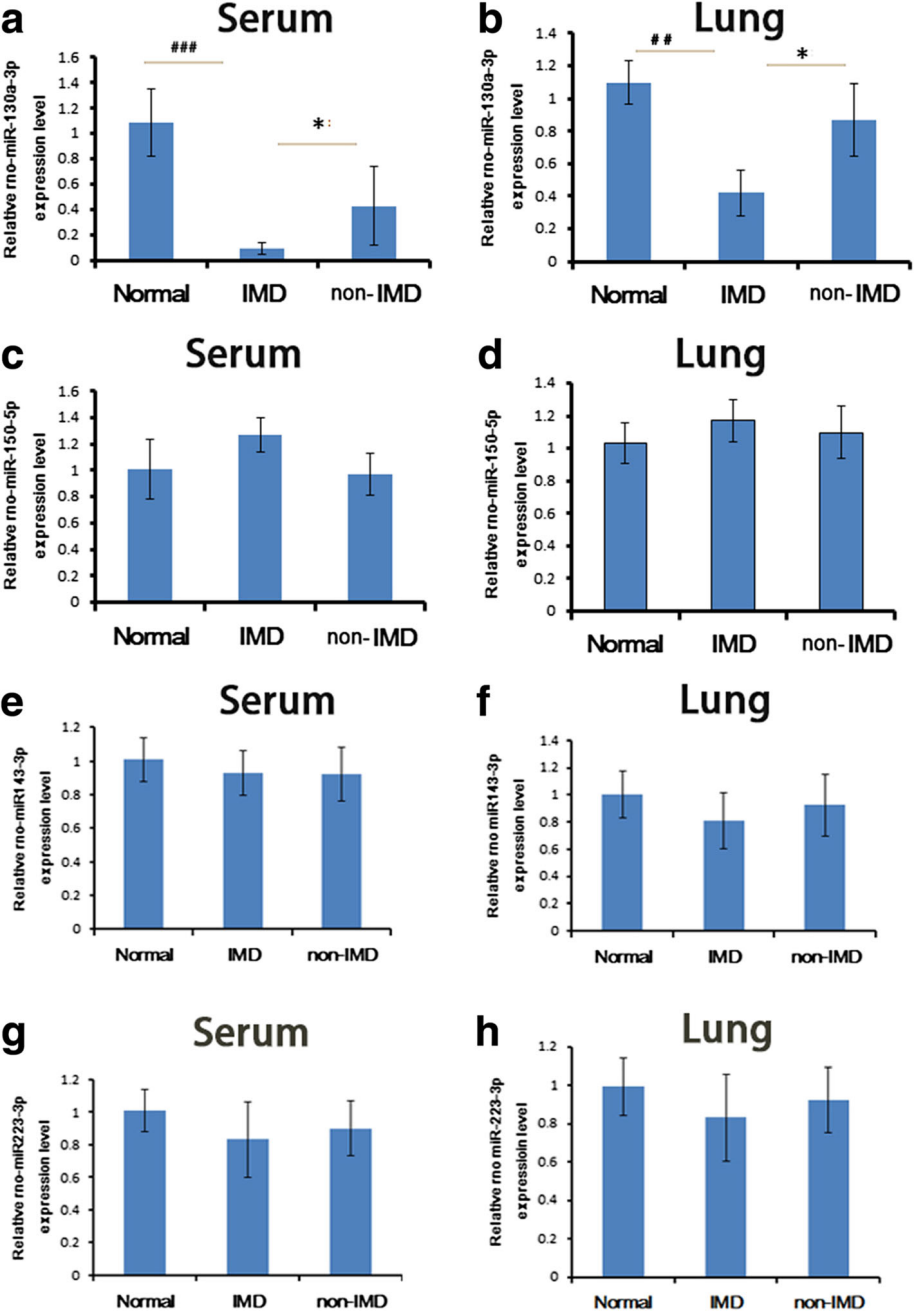
Validation of miR130a-3p, miR-150-5p, miR-143-3p, and miR-223-3p in serum and lung tissues by qRT-PCR**.** Serum (**a**) and lung (**b**) levels of miR-130a-3p in normal, immune disturbance (IMD), and non-IMD rats. Serum (**c**) and lung (**d**) levels of miR-150-5p in normal, IMD, and non-IMD rats. Serum (**e**) and lung (**f**) levels of miR-143-3p in normal, IMD, and non-IMD rats. Serum (**g**) and lung (**h**) levels of miR-223-3p in normal, IMD, and non-IMD rats. *N* = 6, *P < 0.05 for comparison between IMD and non-IMD, ^##^*P* < 0.01 and ^###^*P* < 0.001 for comparison between IMD and normal rats

To validate the reliability of the microarray result, 4 of the 13 differentially expressed miRNAs were selected for validation by qRT-PCR in the lung and serum of normal, IMD, and non-IMD rats. The data showed that the serum and lung levels of miR-130a-3p were significantly downregulated in IMD rats compared with those in normal and non-IMD rats (*P* < 0.001) (Fig. 3a, b). In contrast, there was no significant difference in the serum and lung levels of miR-150-5p, miR-143-3p, and miR-223-3p of among the normal, IMD, and non-IMD rats (*P* > 0.05, Fig. 3c–h).

### Functional categories of downregulated miRNAs in IMD rat serum

**Fig. 4:**
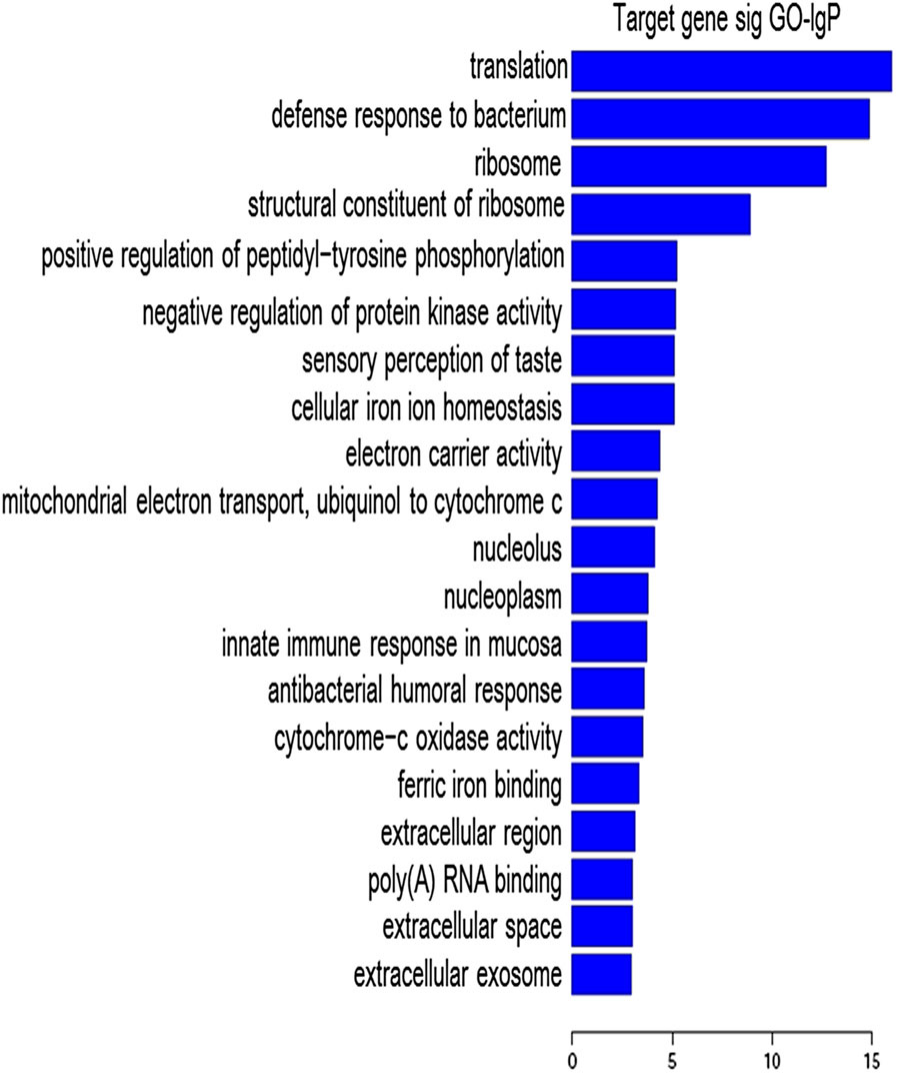
Gene Ontology (GO) functional categories of the seven downregulated miRNAs. The vertical axis showed the GO category, and the horizontal axis showed the enrichment of GO

According to GO analysis of biological processes, the seven significantly downregulated miRNAs were classified into different functional categories (Fig. 4). All of these GOs showed an increased enrichment in each category by these miRNAs. The top five GO functional categories for the downregulated miRNAs were as follows: transport, defense response to bacterium, ribosome, structural constituent of ribosome and positive regulation of peptidyl-tyrosine phosphorylation.

### Downregulated miRNA-associated regulatory networks

**Fig. 5:**
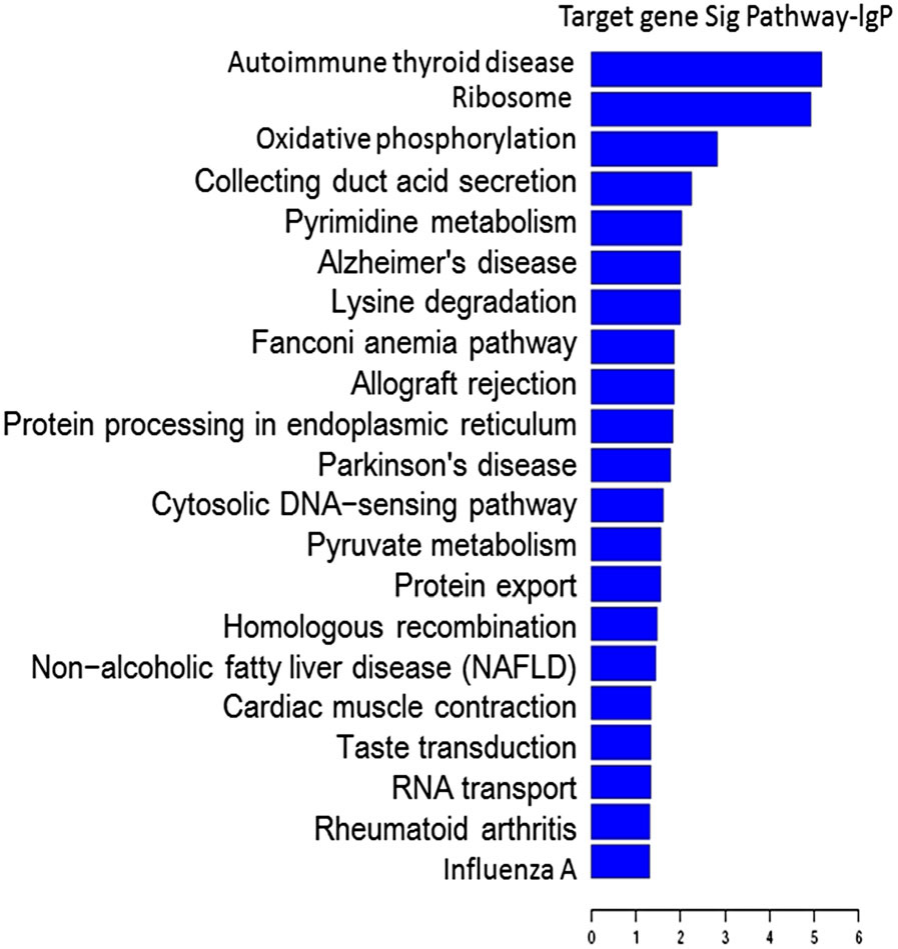
Pathway analysis of the differentially downregulated miRNAs. The vertical axis showed the pathway categories and the horizontal axis showed the degrees of the enrichment of the pathways

The pathways associated with the differentially expressed miRNAs were determined by KEGG pathway analysis. The most significant pathways of the downregulated miRNAs concerned autoimmune thyroid disease, ribosome, oxidative phosphorylation, collecting duct acid secretion, and pyrimidine metabolism (Fig. 5).

### The potential target genes of miR-130a-3p

**Fig. 6:**
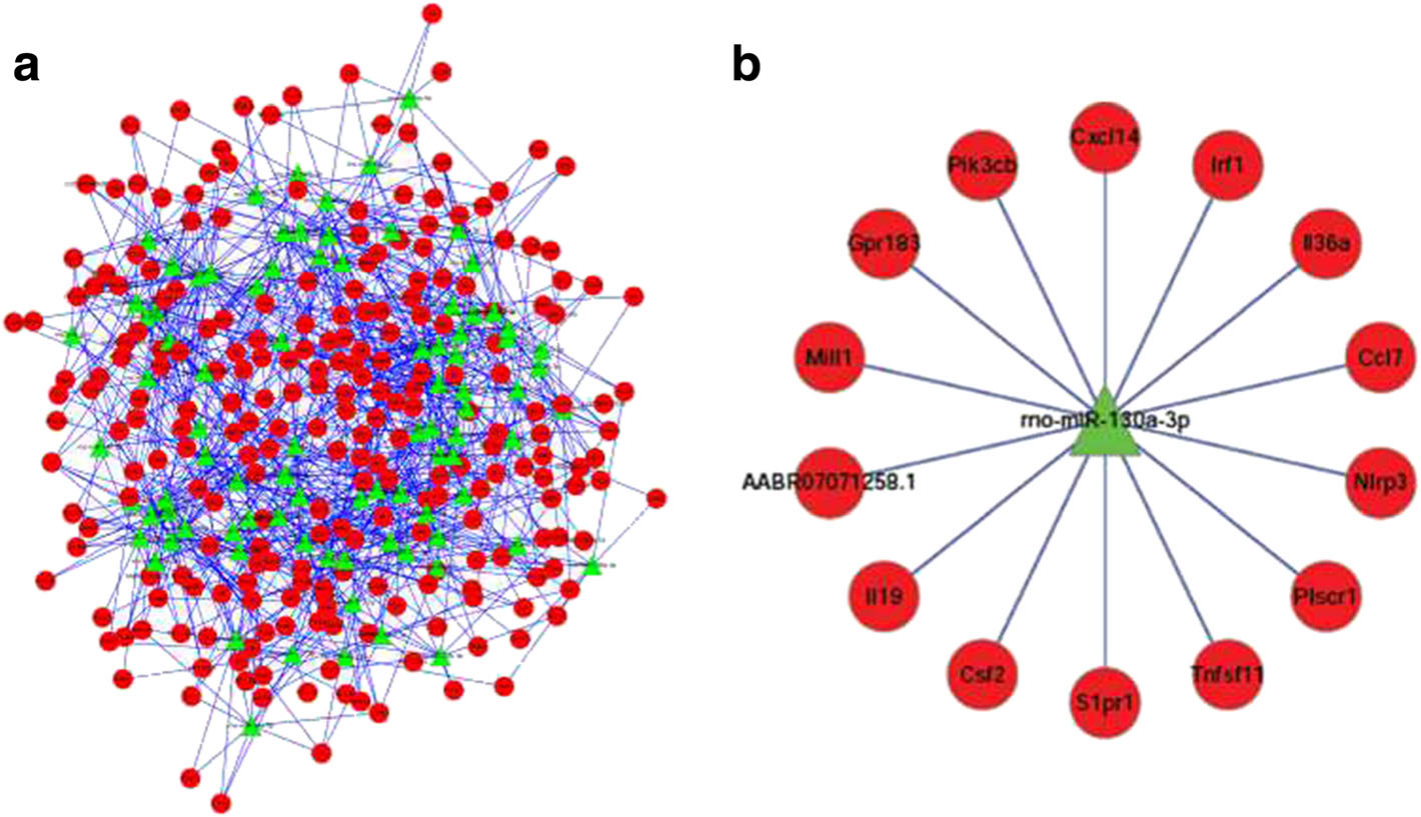
miRNA–mRNA network in immune system. **a** Green box nodes represented miRNAs, and red cycle nodes represented mRNAs. **b** Right panel showed the target mRNA network of miR-130a-3p in immune system

Furthermore, to illustrate the role of miR-130a-3p, significantly decreased in the serum and lung tissues of IMD rats in comparison to normal and non-IMD rats as validated by RT-PCR, in the regulation of immune system in IMD rats, a miRNA-mRNA network was suggested. In total, 235 mRNAs which may differentially regulate the miR-130a-3p-targeted mRNAs were included in the network (Fig. 6a). Particularly, 14 mRNAs as the potential target genes of miR-130a-3p, such as *IRF1*, *S1PR1*, *Ccl7*, *Pik3cb*, *and Cxcl14*, were supposedly associated with immune system (Table 3, Fig. 6b).

**Table 3:** Target mRNA network of miR-130a-3p in the immune system

Target gene	Representative transcript	Species	Gene name
MILL1	ENSRNOT00000035286	*Rattus norvegicus*	MHC I-like leukocyte 1(Mill1)
PLSCR1	ENSRNOT00000010689	*Rattus norvegicus*	Phospholipid scramblase 1(Plscr1)
GPR183	ENSRNOT00000034560	*Rattus norvegicus*	G protein-coupled receptor 183(Gpr183
CXCL14	ENSRNOT00000016009	*Rattus norvegicus*	C-X-C motif chemokine ligand 14(Cxcl14)
IRF1	ENST00000245414.4	*Rattus norvegicus*	Interferon regulatory factor 1(Irf1)
NLRP3	ENSRNOT00000086710	*Rattus norvegicus*	NLR family, pyrin domain containing 3 (Nlrp3)
PIK3CB	ENSRNOT00000022179	*Rattus norvegicus*	Phosphatidylinositol-4,5-bisphosphate 3-kinase, catalytic subunit beta(Pik3cb)
S1PR1	ENSRNOT00000018318	*Rattus norvegicus*	Sphingosine-1-phosphate receptor 1(S1pr1)
CSF2	ENSRNOT00000032333	*Rattus norvegicus*	Colony stimulating factor 2(Csf2)
CCL1	ENSRNOT00000031360	*Rattus norvegicus*	C-C motif chemokine ligand 1(Ccl1)
TNFSF11	ENSRNOT00000049056.4	*Rattus norvegicus*	Tumor necrosis factor superfamily Member 11(Tnfsf11)
IL19	ENSRNOT00000035044	*Rattus norvegicus*	Interleukin 19(Il19)
IL36A	ENSRNOT00000007540	*Rattus norvegicus*	Interleukin 36, alpha(Il36a)

### S1PR1 and IRF1 protein were upregulated in IMD rat lung

**Fig. 7:**
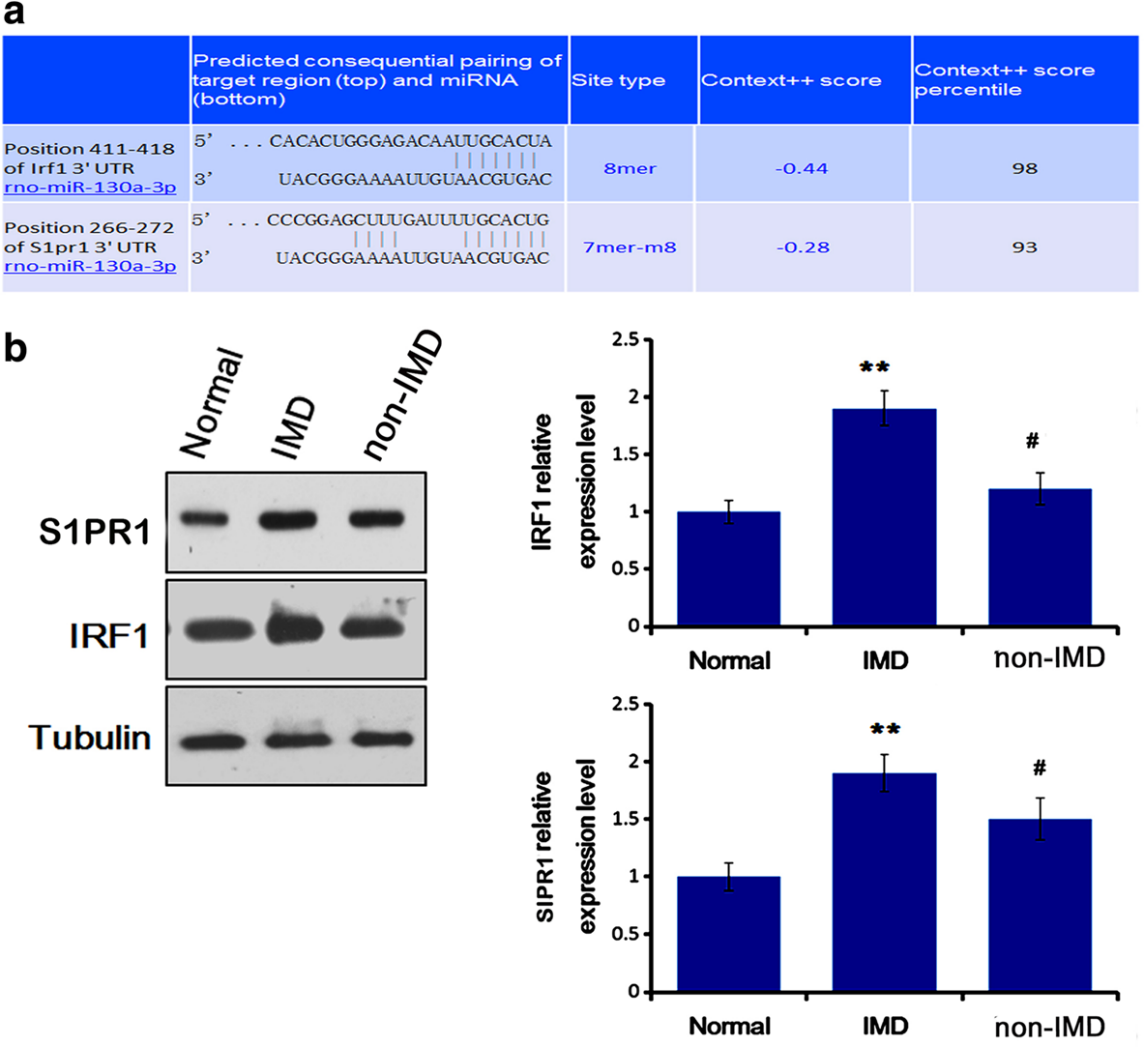
MiR-130a-3p inhibited the SIPR1 and IRF1 expression in rat lung. **a** Prediction of *SIPR1* and *IRF-1* as the potential target genes of miR-130a-3p. **b** Western blotting for the protein expression SIPR1 and IRF1 in rat lung. *n* = 6, **P < 0.01 for comparison between immune disturbance (IMD) and normal rats, and ^#^*P* < 0.05 for comparison between IMD and non-IMD rats

Using TargetScan software, *S1PR1* and *IRF1* were predicted as the potential target genes of miR-130a-3p (Fig. 7a). The expression level of S1PR1 and IRF1 in rat lung tissues was determined by Western blotting. The result showed that the lung S1PR1 and IRF-1 expression was significantly increased in IMD rats compared with that in normal and non-IMD rats (*P* < 0.05, Fig. 7b).

## Discussion

Hip fracture frequently occurs in the elderly, with a high incidence of morbidity and mortality [2]. Reportedly, hip fracture causes systemic pro-inflammatory response [2, 6, 7, 9–11]. Particularly, patients with hip fracture have significantly increased serum levels of inflammatory cytokines (e.g., TNF-α, IL-6, IL-10) compared with healthy controls [2, 6, 7]. In this study, we determined the serum levels of TNF-α and IL-10 in normal versus hip fracture rats. Elderly rats were randomly divided into normal or hip fracture groups. To avoid any potential resultant inflammatory response from blood collecting, the hip fracture rat group was classified into three subgroups for collecting blood at 24, 48, or 72 h, respectively, after the hip injury. Our result showed that in elderly hip fracture rats, the serum levels of TNF-α and IL-10 were almost significantly increased compared with healthy controls, which was consistent with those previously reported [2, 6, 7].

TNF-α as a pro-inflammatory cytokine is mainly produced by monocytes, macrophages, initial hemorrhages, and necrosed tumor tissues [16]. IL-10 is a cytokine that downregulates the immune response and inflammation by suppressing the expression of pro-inflammatory cytokines and downregulating important cell surface molecules such as MHC class II molecules [17]. There is a consensus as to a protective effect of IL-10 on the inflammatory action of TNF-α during systemic inflammatory response syndrome [18]. Low TNF-α/IL-10 ratio, characterized by IL-10 superiority, indicates a status of immunosuppression in IMD [8]. In this study, 13.3% (6 of 45) of hip fracture rats were dead or became moribund at 72 h after injury, which was simultaneously accompanied by a significant decrease of TNF-α/IL-10 ratio compared with non-IMD hip fracture and normal rats. This implies an immunosuppression status in these dead or moribund rats (IMD) with hip fracture.

As small non-coding 22-nucleotide RNA molecules, miRNAs involve in post-transcriptional regulation either by mRNA cleavage and degradation or by repressing the translation of mRNA into proteins [19]. MiRNA control has emerged as a critical regulatory principle in the mammalian immune system [14], and it has been shown that miRNAs may be used as potential biomarkers for the diagnosis of various human diseases [20]. A recent study showed that the serum levels of miR-122-5p, miR-125b-5p and miR-21-5p were significantly upregulated in patients with bone fracture in comparison with healthy controls [21], implying the potential values of these miRNAs as biomarkers for bone fracture. However, until now, there have no miRNA profiling studies on hip fracture. In this study, we screened the differentially expressed miRNAs between the normal, IMD, and non-IMD elderly hip fracture rats by using miRNA microarray and further confirmed that the miR-130a-3p levels of the serum and lung tissue in IMD rats were both significantly reduced compared with those in normal and non-IMD rats using qRT-PCR. This implies that miR-130a-3p may be used as a potential biomarker for the IMD related to hip fracture in the elderly.

To further understand the role of miR-130a-3p in the regulation of immune response in aged IMD rats, a miRNA-mRNA network was suggested, including 14 mRNAs (e.g., S1PR1 and IRF-1 genes) related to immune system as the potential target genes of miR-130a-3p. Target prediction showed that S1PR1 and IRF-1 genes are potential targets of miR-130a-3p. Since reportedly elderly hip fracture often accompanies with postoperative liver and lung dysfunction [2, 6, 7], we selectively observed the expression of miR-130a-3p and protein of S1PR1 and IRF in lung tissue. Sphingosine 1-phosphate (S1P) is a major mediator of T cell lymphoid traffic, tissue migration and proliferation, and cytokine secretion [22]. S1PR1 as a receptor of S1P restrains thymic development, and peripheral number and suppressive functions of T regulatory cells.^23^ Increase of S1PR1 in CD4^+^ T cells promotes STAT3 activation [23], and STAT3 is the main downstream molecular target for IL-6R and IL-10R signaling and promotes IL-10 while inhibits IL-12 production [24]. Conversely, IL-10-induced STAT3 activity in macrophages leads to impaired antigen-specific T cell responses [25]. Therefore, S1PR1, as an immunosuppresive factor, may induce the production of IL-10 via STAT3 signaling. IRF-1 is a nuclear transcription factor crucial to inflammation, immunity, cell proliferation, and apoptosis [26]. IRF1 and the transcription factor complex ISGF3 (including STAT1, STAT2, and p48) mediate the upregulation of IL-10 expression by type I interferon (IFN) and IFN-α. Activation of IRF1 and STAT3 in IFN-α-stimulated human monocytes initially contributes to the production of IL-10, pro-inflammatory cytokines, etc. by low level autocrine IFN-α-mediated signaling [27, 28]. Therefore, IRF1 may also participate in the regulation of the IL-10 production. In this study, we found the lung expression of SIPR1 and IRF1, both related to the production of IL-10, were significantly increased in IMD rats, suggesting that the reduction of miR-130a-3p may lead to increased expression of SIPR1 and IRF1, which mediates the production of IL-10. In addition, a previous study reported that miR-130a directly targeted the 3′-UTR of TNF-α and repressed its translation [29]. Consistently, this study revealed that decrease of miR-130a-3p promotes the production of the pro-inflammatory factor TNF-α in hip fracture rats.

In this study, through the target prediction of miR-130a-3p using bioinformatics tool and the measurement of the lung levels of miR-130a-3p, S1PR1, and IRF1, we supposed that S1PR1 and IRF are the targets of miR-130a-3p. The next study will be carried out to determine the direct control of miR-130a-3p toward S1PR1 and IRF genes.

## Conclusions

This study demonstrates that hip fracture-induced IMD, characterized by reduced TNF-α/IL-10 ratio, in aged rats may be attributable to the downregulation of miR-130a-3p and the upregulation of target *S1PR1* and *IRF1*. Our experimental results may aid in the understanding of the molecular mechanisms underlying the roles of miRNAs in the pathogenesis of IMD related to elderly hip fracture. This study might provide a new potential biomarker with the diagnostic and prognostic values and a potential therapeutic target for hip fracture-induced IMD in the elderly as well.

## Abbreviation

GO: Gene ontology
IL-10: Interleukin-10
IMD: Immune disturbance
IRF1: Interferon regulatory factor-1
KEGG: Kyoto encyclopedia of genes or genomes
miRNA: MicroRNAs
qRT-PCR: Quantitative real-time reverse transcription polymerase chain reaction
S1P: Sphingosine 1-phosphate
S1PR1: Sphingosine-1-phosphate receptor 1
SD: Sprague Dawley
TNF-α: Tumor necrosis factor-α
